# Shared metabolism between a bacterial and fungal species that reside in the human gut

**DOI:** 10.1073/pnas.2504785122

**Published:** 2025-08-25

**Authors:** Haley Gause, Alexander D. Johnson

**Affiliations:** ^a^TETRAD Graduate Program, Department of Biochemistry and Biophysics, University of California, San Francisco, CA 94158; ^b^Department of Microbiology and Immunology, University of California, San Francisco, CA 94158

**Keywords:** gut microbiome, *Candida albicans*, *Enterococcus faecalis*, RNA-seq

## Abstract

The fungal species *Candida albicans* and the bacterium *Enterococcus faecalis* are members of the human gut microbiome. To explore the range of interactions between these two species, we utilized dual RNA-sequencing to transcriptionally profile both *C. albicans* and *E. faecalis* during coculture (compared with monoculture controls) under two conditions: 1) an in vitro setting that mimics certain features of the gut environment and 2) a gnotobiotic mouse gut model. RNA-seq analysis revealed a large number of gene expression changes induced by one species in the presence of the other. More specifically, both species highly upregulate citrate-metabolizing genes during coculture: *C. albicans* upregulates *CIT1* (citrate synthase) which produces citrate, while *E. faecalis* upregulates its *cit* operon, which breaks down citrate. In vitro analysis showed directly that citrate cross-feeding (production of citrate by *C. albicans* and breakdown by *E. faecalis)* enhances growth of *E. faecalis*. A main byproduct of citrate metabolism in *E. faecalis* is formate, a short chain fatty acid toxic to fungi. Our RNA profiling revealed that *C. albicans* upregulates three formate dehydrogenases (FDHs) during coculture; we show that the FDH genes confer a growth advantage to *C. albicans* when *E. faecalis* (or simply formate) is present. These findings reveal a metabolically driven cycle between *C. albicans* and *E. faecalis* in the mouse gut and in vitro, where cross-feeding of citrate and detoxification of formate facilitates the growth of both species when they are cultured together.

## Significance

The gut microbiome is a complex mixture of species, and it has been difficult to study detailed interactions between its different components. Here, we use two common colonizers of the gut, *Candida albicans* and *Enterococcus faecalis*, to study how a bacterial and fungal species interact. Using dual RNA-sequencing both in the gut of germ-free mice and in vitro, we measured drastic changes in the gene expression profile of both species during coculture compared to growth alone. In follow-up experiments, we uncovered a metabolic sharing cycle between the two species that is both mutually beneficial and antagonistic.

The adult human gut microbiome is a complex community of microorganisms; the interactions among them shape large ecological and metabolic networks (1–3). This microbial community also interacts with the host to support numerous important aspects of human health, including maintenance of the gut epithelial layer and priming of the immune system (4, 5). In addition, a diverse gut microbiome protects against perturbations to community homeostasis and the expansion of opportunistic microbial species (6). Blooms of these pathobionts are increasingly associated with many gastrointestinal, neurological, and immune disease states (6, 7).

While the gut microbiome is primarily composed of bacterial species, it also includes a diverse array of other microorganisms, including archaea and fungi, albeit at lower levels of colonization (8). Multiple studies have reported fungal burdens of healthy humans around 10^1^ to 10^3^ CFU per gram of feces compared to bacterial levels, which reach 10^11^to 10^12^ CFU per gram (9, 10). However, fungi are almost 100 times larger than bacteria by biomass, and their presence has been shown to significantly modulate the bacterial proteome within the gut microbiome (11, 12). These results suggest that, despite their relatively low cell numbers, fungi can exert a considerable influence in the gut environment.

One of the most common fungal colonizers of the gut microbiome is *Candida albicans*. This yeast species has been estimated to be present in 75% to 95% of the adult human population (13–15). In addition, *C. albicans* is one of the first colonizers of the neonate gut microbiome and is estimated to colonize up to 96% of all infant guts (10). Although *C. albicans* is present in comparatively low numbers in a healthy gut microbiome, dysbiosis can lead to blooms of resident *C. albicans* as high as 10^5^ to 10^7^ CFU per gram of feces (16). These fungal blooms have been observed to precede the onset of intestinal inflammation, epithelial barrier dysfunction, and translocation of *C. albicans* from the gut to bloodstream where it can cause systemic infection with mortality rates as high as 40% (16–18).

In multiple studies of mouse models and human patients, blooms of *C. albicans* in dysbiotic gut microbiomes were associated with increases in the levels of the bacterium *Enterococcus faecalis* (16, 19, 20). As with *C. albicans*, this gram-positive firmicute is also found at relatively low concentrations in the healthy adult gut, can bloom to high numbers in periods of dysbiosis, and can translocate across compromised gut barriers to cause fatal bloodstream infections (21, 22). In addition, like *Candida* species, *E. faecalis* is among the most common colonizers of the preterm infant gut microbiome. In a recent metagenomic survey of preterm infant gut microbiomes, *Candida* species and *E. faecalis* were the primary microbial species present in a set of neonates (23). The correlation and behavior of *C. albicans* and *E. faecalis* in the gut suggests an interaction that can significantly impact host health.

Previous studies have documented interactions between *E. faecalis* and *C. albicans*: *E. faecalis* has been shown to reduce the formation of hyphae in *C. albicans*, and *Caenorhabditis elegans* colonized with both *C. albicans* and *E. faecalis* lead to a decrease in invasive growth of the microbes beyond the gut that is typically observed when worms were colonized with either species alone (24, 25).

In this study, we describe the changes in gene expression when *C. albicans* and *E. faecalis* are cultured together compared to separate growth. By pursuing leads from this analysis, we demonstrate a shared metabolic cycle in which *C. albicans* produces citrate, which is broken down by *E. faecalis*, generating formate as a byproduct. This formate, in turn, induces the expression of formate-detoxifying enzymes in *C. albicans*. We propose that this type of metabolic cycle exemplifies the pairwise metabolic interactions between fungal and bacterial species in the gut.

## Results

### Transcriptional Response of *C. albicans* to *E. faecalis* In Vitro.

**Fig. 1.**
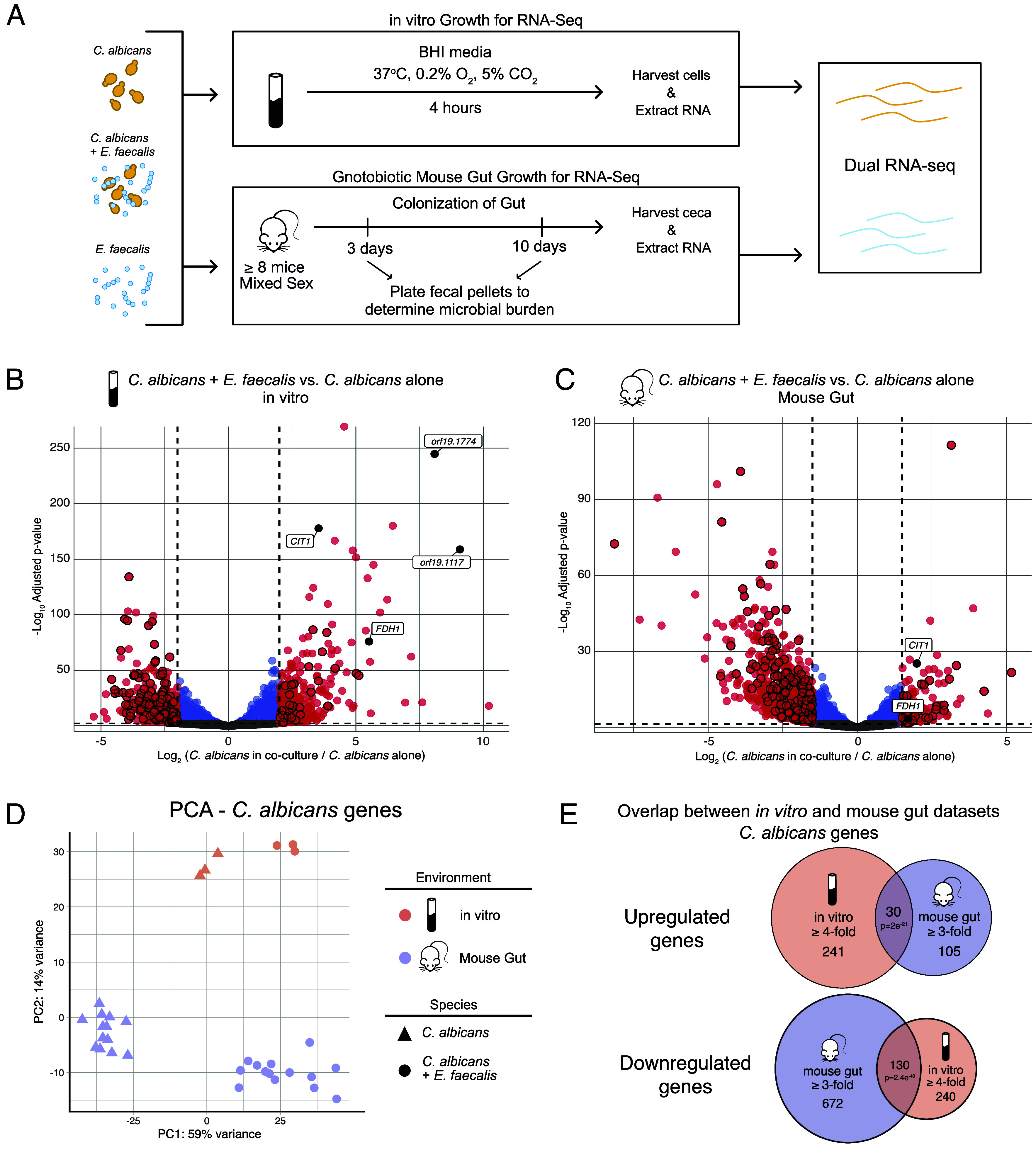
Transcriptional profiling of *Candida albicans* gene expression in response to *Enterococcus faecalis* in vitro and in the mouse gut. (*A*) Schematic of the dual RNA-seq experiment, where *C. albicans* and *E. faecalis* were grown either in monoculture or coculture under in vitro conditions or in the gut of gnotobiotic C57BL/6 mice. (*B* and *C*) Volcano plots showing *C. albicans* gene expression changes when cocultured with *E. faecalis* compared to monoculture in vitro (*B*) or in the mouse gut (*C*). Each point represents a single *C. albicans* gene. Induced genes are on the *Right*, and repressed genes are on the *Left*. Genes are color-coded as follows: red: genes with > 4 (in vitro) or 3 (mouse) fold-change in expression (adjusted *P*-value < 0.05); blue: significant change in expression, but < 4 (in vitro) or 3 (mouse) fold-change in expression; gray: nonsignificant changes. Black-outlined dots highlight genes significantly up- or downregulated in both in vitro and mouse gut conditions. Labeled genes include *CIT1* and the three formate dehydrogenase (*FDH*) genes. (*D*) Principal component analysis (PCA) plot of RNA-seq results, illustrating gene expression patterns for *C. albicans* grown alone (triangles) or with *E. faecalis* (circles) under either in vitro (peach) or mouse gut (purple) conditions. Each dot represents a sample, with clustering indicating intragroup similarity and separation reflecting intergroup differences. (*E*) Venn diagrams depicting the overlap of upregulated (*Top*) and downregulated (*Bottom*) *C. albicans* genes induced by *E. faecalis* (black-outlined genes in *B* and *C*). Statistical significance of the overlap is determined by a hypergeometric test (*N* = 6,200 genes).

To determine how *C. albicans* responds to the presence of *E. faecalis* (and vice versa), we employed a dual RNA-seq approach. Total RNA was collected from monocultures of *C. albicans* and *E. faecalis* and cocultures of both species grown in Brain Heart Infusion (BHI) media for 4 h in low-oxygen (0.2% O_2_) (Fig. 1*A*). BHI medium was chosen for this study as it is a rich, complex media that is a suitable growth medium for *C. albicans* and *E. faecalis* individually and has been shown to maintain a diversity of microbes isolated from the human gut in vitro (26, 27). 0.2% oxygen was used in this study because the lumen of the human colon is known to have low oxygen content (0.4 to 1%) (28), and 0.2% oxygen allows ample growth of both *C. albicans* and *E. faecalis* in laboratory settings. After rRNA depletion and cDNA library generation (see *Materials and Methods* for details), coculture reads were mapped onto a concatenated *C. albicans*-*E. faecalis* genome simultaneously to decrease incorrect cross-species mapping, while monoculture samples were mapped only to their own respective genome. After trimming and quality filtering, an average of 2.5 × 10^7^ reads were mapped onto the genome(s) per sample. In *C. albicans* monocultures, 86% of those reads were assigned to annotated features, while 9% of the mapped reads in the *C. albicans*-*E. faecalis* coculture were assigned to *C. albicans* features.

We observed that *C. albicans* undergoes a large number of high magnitude transcriptional changes in the presence of *E. faecalis*. We defined differentially expressed genes as those with a minimum four-fold change in gene expression (adjusted *P*-value (*P*adj) < 0.05) between *C. albicans* grown in the presence of *E. faecalis* compared to *C. albicans* grown by itself. This high threshold was selected to highlight the most significant changes. Even so, we observed 271 genes upregulated at least four-fold and 370 genes downregulated at least four-fold (Fig. 1*B* and Dataset S1). Using an even higher threshold, we identified 98 genes that were upregulated and 98 genes that were downregulated at least eight-fold in the presence of *E. faecalis* under these in vitro conditions. We focused our attention on a small number of these large changes and followed these up by direct experimentation as described in later sections.

### Transcriptional Response of *C. albicans* to *E. faecalis* in the Gut Microbiome.

Previous work has established that the *C. albicans* gene expression profile in vitro and in the mammalian gut are vastly different (29). To account for the impact of the host in our mixed microbial cultures, we transcriptionally profiled *C. albicans* and *E. faecalis* in the gut of mono- or cocolonized gnotobiotic mice. A minimum of eight germ-free C57BL/6 mice were orally gavaged with either *C. albicans* alone, *E. faecalis* alone, or with a mix of both species (1:100 yeast:bacteria ratio) (Fig. 1*A*). Mice were colonized for 10 d and colonization was monitored by plating fecal pellets at three and 10 d. At day 10, colonization levels of *E. faecalis* were not significantly different between mono- and cocolonized mice; however, the burden of *C. albicans* in the cocolonized mice was significantly lower at day 10 compared to mice colonized with *C. albicans* alone (*SI Appendix*, Fig. S1), indicating that the colonization of *C. albicans* is reduced in the presence of *E. faecalis*.

Total RNA was extracted from the cecal contents of mice 10 d after gavage and used to generate cDNA libraries following the same protocol as used for the in vitro cDNA libraries. After filtering low-quality reads, an average of 5.5 × 10^7^ reads per sample were used as input for genome(s) alignment, and 2.8 × 10^7^ reads per sample (52%) were uniquely mapped onto the genome(s). The low mapping percentage is unsurprising given the high complexity of the cecum content samples. 75% and 3% of mapped reads were assigned to *C. albicans* features in mono- and cocolonized mice, respectively.

To determine how similar the in vitro *C. albicans* transcriptional profile was to that of the mouse gut, principal component analysis (PCA) was performed on all samples. PCA revealed that the first principal component (PC1) was driven by whether *C. albicans* was grown in the presence or absence of *E. faecalis*; that is, a majority (59%) of the variance seen across all samples can be explained the presence of *E. faecalis* on *C. albicans* in coculture regardless of the environment (Fig. 1*D*). The second principal component (PC2), representing the second largest source of variance (14%), separates the samples based on the environment in which they are grown, the mouse gut or in vitro. The PCA revealed a tight clustering within samples grown in the same environment and condition (e.g. *C. albicans* grown alone in the mouse gut) and distinct separation between different sample groups. This analysis also demonstrates a high reproducibility of *C. albicans* expression from mouse to mouse and clearly shows that the presence of *E. faecalis* has major effects on *C. albicans* both in vitro and in vivo.

For mouse colonized samples, differentially expressed genes were defined as *C. albicans* genes with at least three-fold significant change (*P*adj < 0.05) in expression in the presence of *E. faecalis* compared to mice colonized with *C. albicans* alone. We chose this lower threshold (compared to the in vitro comparisons) because the sequencing depth in the mouse experiments is lower than the in vitro samples. As we observed in vitro, the gene expression of *C. albicans* in the mouse gut is significantly impacted by the presence of *E. faecalis*; 135 and 802 genes are significantly up- and downregulated at least three-fold, respectively, in the presence of *E. faecalis* compared to colonization by *C. albicans* alone (Fig. 1*C* and Dataset S1).

When comparing the response of *C. albicans* to *E. faecalis* in the mouse gut to that in vitro, there is significantly more overlap than one would expect by chance (*P* < 2e^−21^). 39 genes are upregulated and 130 genes are downregulated by *C. albicans* both in vitro and in the mouse gut in the presence of *E. faecalis* (Fig. 1*E* and Dataset S1). We note that more than half of the 34 upregulated genes are currently uncharacterized in *C. albicans* and await further study. The 130 downregulated genes are overrepresented for pathways including glycolysis and gluconeogenesis, and acetyl-CoA generation from pyruvate.

### Transcriptional Profiling of *E. faecalis* During Growth with *C. albicans*.

As our RNA extraction protocols and library preparations were designed to sequence RNA from both *C. albicans* and *E. faecalis*, we could therefore simultaneously examine how *E. faecalis* responds to *C. albicans* in the same cocultures discussed above. As with *C. albicans*, drastic changes were observed in *E. faecalis* in response to growth with *C. albicans* in vitro: 249 genes are upregulated and 153 genes are downregulated at least four-fold in the presence of *C. albicans* (*SI Appendix*, Fig. S2*A* and Dataset S2). In the mouse gut, we observed little to no significant effect of *C. albicans* on *E. faecalis* gene expression (*SI Appendix*, Fig. S2*B*). This likely reflects our experimental setup, as the mouse experiments were designed primarily to capture the response of *C. albicans* to *E. faecalis*; the gavage ratio and the point of euthanasia were selected to produce a large excess (10^5^) of *E. faecalis* relative to *C. albicans* (*SI Appendix*, Fig. S1). At these ratios, it is likely *C. albicans* is not abundant enough to induce extensive changes in gene expression in *E. faecalis*.

In the following sections, we develop a set of hypotheses based on the RNA-seq data and test them experimentally.

### *E. faecalis* Induces the Upregulation of *C. albicans* Citrate Synthase During Coculture.

**Fig. 2.**
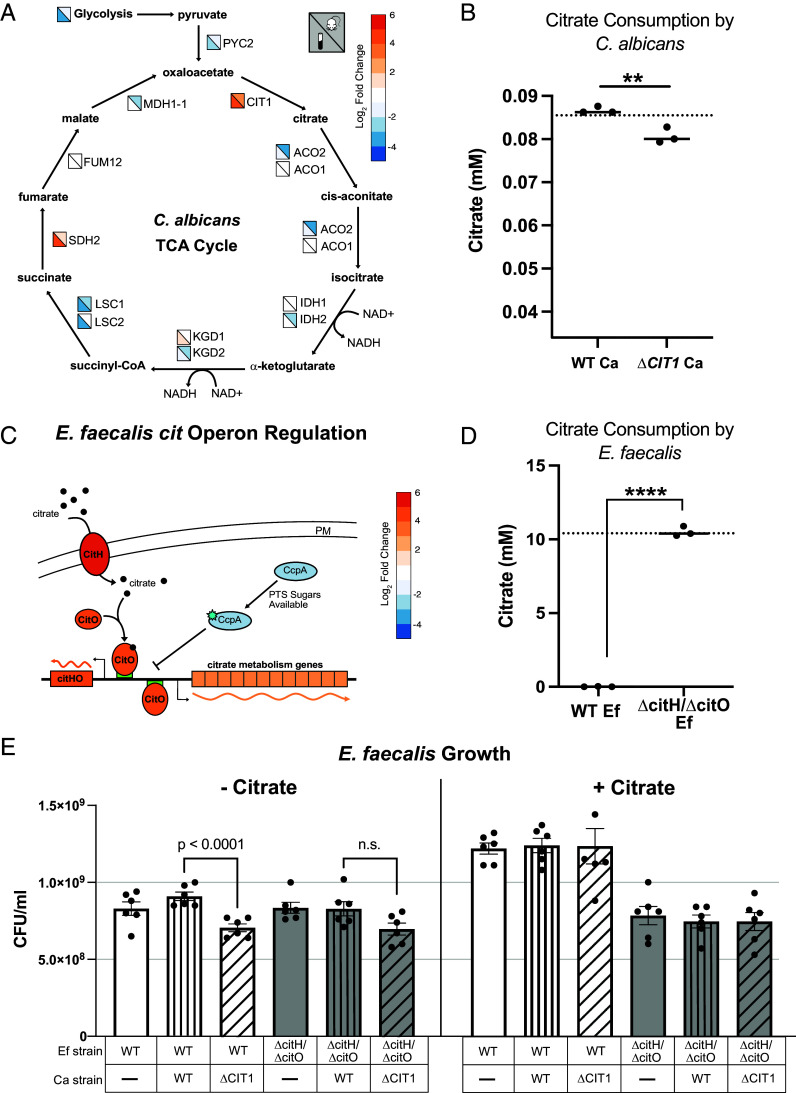
*C. albicans* and *E. faecalis* upregulate citrate production and consumption in coculture (*A*) Diagram depicting the TCA Cycle in *C. albicans*. Reactions indicated by arrows are labeled with the enzyme responsible for that reaction. Boxes next to enzyme names indicate Log_2_ fold changes in expression in *C. albicans* when grown with *E. faecalis* compared to growth alone as determined by RNA-seq. The upper right triangle in each box represents the fold-change in the mouse gut, the lower left triangle in each box represents the fold-change in vitro. (*B*) Extracellular citrate concentrations in 24 h cultures of either WT *C. albicans* or an isogenic ∆*cit1* deletion strain of *C. albicans*. The dotted line represents the citrate concentration of blank BHI(-glu) media. The *y*-axis starts at the lower limit of detection for assay. Data are shown as individual points (n = 3) with a line indicating the mean; statistical significance was determined using an unpaired Student’s *t* test (*P* = 0.0065). (*C*) Diagram depicting regulation of the *cit* operon in *E. faecalis*, adapted from Martino et. al (30) Color of proteins and operon gene blocks represent Log_2_ fold change in *E. faecalis* gene expression in the presence of *C. albicans* compared to *E. faecalis* alone. (*D*) Extracellular citrate concentration in 24 h cultures of WT *E. faecalis* or an isogenic *∆citH/∆citO* deletion strain of *E. faecalis*. The dotted line represents the citrate concentration of blank BHI(-glu,+cit) media. Data are shown as individual points (n = 3) with a line indicating the mean; statistical significance was determined using an unpaired Student’s *t* test (*P* < 0.0001). (*E*) Barplot representing the concentration (CFU/mL) of *E. faecalis* in monoculture or coculture with *C. albicans* after 24 h of growth in BHI(-glu) (*Left*) or BHI(-glu) with 0.2% added citrate (BHI(-glu,+cit)) (*Right*). Data are shown as individual points (n = 6) with bars indicating the mean, and error bars representing ± SEM; statistical significance was determined using a one-way, paired ANOVA (within each media group), with Šidák’s multiple comparison test (n.s. *P*-value = 0.33).

One of the highest upregulated *C. albicans* genes in response to *E. faecalis*, both in the mouse gut and in vitro conditions, is citrate synthase (*CIT1*) (Fig. 1 *C* and *D*). Cit1 is the first enzyme of the TriCarboxylic Acid (TCA) cycle and catalyzes the addition of acetyl-CoA to oxaloacetate to produce citrate. While this enzyme is highly induced in the presence of *E. faecalis* (12-fold upregulated in vitro, four-fold in the mouse gut), the additional enzymes of the TCA cycle do not show a similar pattern of upregulation (Fig. 2*A* and Dataset S3). For example, the TCA enzymes directly downstream of Cit1, Aco1, and Aco2 (which convert citrate to isocitrate), are downregulated in the presence of *E. faecalis.*

Many fungal species are known to secrete citrate; for example, *Aspergillus niger* and *Yarrowia lipolytica* are the main producers of citric acid used in food production (31). The secretion of citrate by fungal species requires both the upregulation of *CIT1* and the downregulation of downstream enzymes of the TCA cycle that would otherwise redirect the flux of citrate through the cycle (32). During coculture with *E. faecalis*, we observed this regulatory pattern in *C. albicans,* suggesting that *C. albicans* secretes citrate in the presence of *E. faecalis* (Fig. 2*A*). Although not to the same degree as the industrial citrate producers, *C. albicans* has also been previously shown to secrete citrate (0.32 to 0.41 mM) (33). In our in vitro system, the extracellular citrate concentration of a WT *C. albicans* culture grown for 24 h in BHI without added glucose [BHI(-glu)] was significantly higher than that produced by an isogenic ∆*cit1* deletion strain, indicating that the *CIT1* gene contributes to citrate secretion in *C. albicans* (Fig. 2*B*). These results indicate that *C. albicans* relies on Cit1-mediated citrate production for its secretion, and that in the presence of *E. faecalis*, this pathway is activated to release citrate into the extracellular environment.

What causes *C. albicans* to upregulate *CIT1* in the presence of *E. faecalis*? We think it is likely that glucose depletion by *E. faecalis* is the signal based on the following observation: *C. albicans* grown in BHI(-glu) expresses *CIT1* at higher levels than in BHI with 0.2% added glucose (*SI Appendix*, Fig. S3). The extent of *CIT1* upregulation by artificial glucose depletion is similar in magnitude to that seen when *C. albicans* is grown in the presence of *E. faecalis* in glucose-replete media.

### Upregulation of *E. faecalis* Citrate Fermentation During Coculture Growth.

One of the most highly upregulated *E. faecalis* operons in response to *C. albicans* in vitro is that encoding the enzymes of citrate fermentation (Fig. 2*C* and Dataset S3). In *E. faecalis*, citrate is metabolized in a two-step process: Citrate lyase complex first converts citrate into oxaloacetate, which is then further converted into pyruvate by oxaloacetate decarboxylase. The 12 enzymes involved in citrate metabolism are encoded within the *cit* operon, which also includes *citO*, a transcriptional activator, and *citH*, a citrate-specific transporter. When citrate is present, it is imported into the cell via CitH, after which it binds and activates CitO, which triggers a positive feedback loop that further induces the expression of *citH* and *citO* (as well as all of the metabolic enzymes) (34). In the presence of *C. albicans*, the entire *cit* operon is upregulated more than 10-fold (Fig. 2*C* and Dataset S3). The transporter *citH* and regulator *citO* are transcribed separately from the main operon and are among the highest upregulated *E. faecalis* genes in the presence of *C. albicans* (45- and 25-fold, respectively). The *cit* operon in *E. faecalis* is repressed by the Carbon Catabolite Repression system, in which the transcriptional repressor CcpA is phosphorylated upon the import of preferential Phosphotransferase System (PTS) sugars, leading CcpA to bind and repress the promoters of alternative carbon metabolic pathways, including the *cit* operon (35). In our RNA-seq experiment, *ccpA* is also downregulated by *E. faecalis* in the presence of *C. albicans,* a change that contributes to an even higher expression of the *cit* operon (Fig. 2*C*).

Previous studies have demonstrated that the upregulation of the *cit* operon in *E. faecalis* corresponds to citrate consumption (34, 36). Consistent with these findings, we observed that in the absence of *C. albicans*, the expression of *citH* and *citO* was significantly increased in BHI(-glu) supplemented with 0.2% citrate [BHI(-glu,+cit)] compared to BHI(-glu) without added citrate (*SI Appendix*, Fig. S4*A*). Moreover, analysis of filter-sterilized supernatants from WT *E. faecalis* cultures grown in BHI(-glu,+cit) showed complete depletion of citrate (Fig. 2*D*). In contrast, an *E. faecalis* ∆*citH/∆citO* deletion mutant cultured under the same conditions was unable to deplete citrate, confirming that the *cit* operon is essential for *E. faecalis* citrate utilization.

Citrate metabolism is known to enhance *E. faecalis* fitness during infection (30). To assess its impact on growth in vitro, we cultured WT and ∆*citH/∆citO E. faecalis* strains in BHI(-glu) with or without 0.2% citrate. In BHI(-glu), both strains exhibited similar growth (*SI Appendix*, Fig. S4*B*). However, in BHI(-glu,+cit), WT reached a higher final optical density (OD) than ∆*citH/∆citO*; the final OD of the ∆*citH*/∆*citO* mutant in BHI(-glu,+cit) was similar to that observed for WT *E. faecalis* grown in BHI(-glu) media, confirming that the increased growth was dependent on both citrate and the *cit* operon. This increase in WT growth scaled with increasing citrate concentrations (*SI Appendix*, Fig. S4*C*). In cultures where glucose was present (BHI), there was little effect of added citrate, supporting previous findings that glucose is a preferred carbon source (35); in the absence of glucose, however, citrate leads to additional growth of *E. faecalis*.

### *C. albicans* Citrate Production Increases Growth of *E. faecalis*.

Given that *C. albicans* citrate production is upregulated in the presence of *E. faecalis* and the *E. faecalis* citrate utilization operon is upregulated in the presence of *C. albicans*, we tested whether citrate production by *C. albicans* can support *E. faecalis* growth. In other words, does *E. faecalis* have a fitness advantage when growing with *C. albicans* due to citrate production from the yeast? To answer this question, we grew cocultures of *C. albicans* and *E. faecalis* in hypoxic conditions in BHI(-glu) without citrate for 24 h. When WT *E. faecalis* was grown in BHI(-glu) with a *C. albicans* strain incapable of making citrate (∆*cit1*), the concentration of *E. faecalis* in a saturated culture was significantly lower than that seen when WT *E. faecalis* was grown with WT *C. albicans (*Fig. 2*E**)*. However, this difference disappeared for the ∆*citH/∆citO E. faecalis* strain, confirming that the growth advantage depends on *E. faecalis* citrate consumption. Adding citrate to the media [BHI(-glu,+cit)] rescued the fitness defect of *E. faecalis* grown with *C. albicans* ∆*cit1*. Taken together, these results show that *E. faecalis* is capable of consuming citrate produced by *C. albicans,* and that this consumption leads to a fitness increase for the bacteria during coculture. While the growth advantage is modest, it is highly reproducible, demonstrating that *CIT1*-intact *C. albicans* provides a growth advantage to *E. faecalis,* as long as the latter has an intact *cit* operon.

### Coculture Induces Upregulation of FDHs in *C. albicans*.

**Fig. 3.**
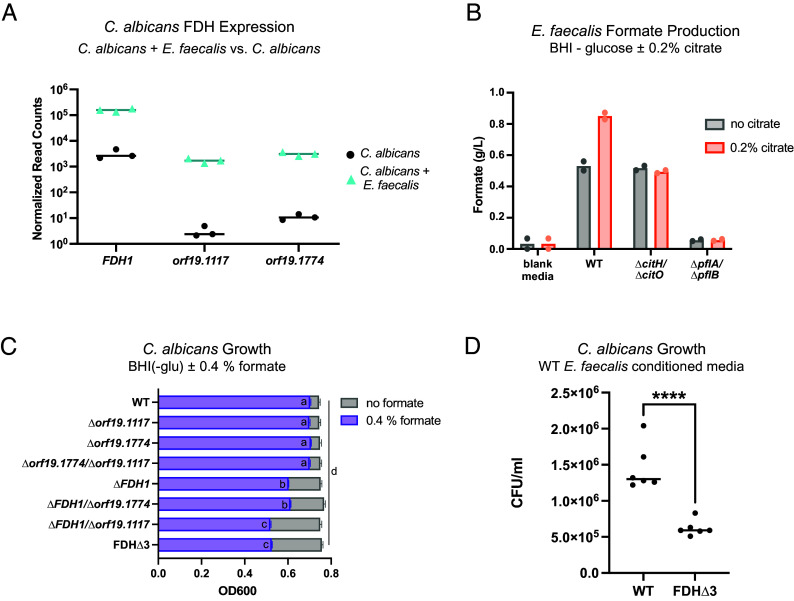
*C. albicans* FDH genes confer a fitness advantage in the presence of *E. faecalis*-derived formate. (*A*) in vitro RNA-seq normalized counts of *C. albicans* FDH genes (*FDH1*, *orf19.1117*, *orf19.1774*) in monocultures (black) and *C. albicans*-*E. faecalis* cocultures (blue). Data are shown as individual points (n = 3) with a line indicating the median. (*B*) Extracellular formate concentration of BHI(-glu) media and 24-h cultures of WT *E. faecalis* and isogenic *∆citH/∆citO* (citrate metabolism deficient) and *∆pflA/∆pflB* (formate production deficient) deletion strains in BHI(-glu) without (gray) or with (orange) added 0.2% citrate. (*C*) Growth of *C. albicans* WT and FDH deletion strains grown in BHI(-glu) without (gray) or with (purple) 4% added formate, shown as the average OD600 in stationary phase (25 to 30 h). Associated growth curves in *SI Appendix*, Fig. S5. Data are shown as bars representing the mean ± SEM. Groups not sharing the same letter are significantly different (*P* < 0.05) based on a two-way ANOVA, with Tukey’s multiple comparisons test. (*D*) Concentration (CFU/mL) of *C. albicans* WT and FDH triple deletion (FDH∆3; ∆*fdh1*/∆*orf19.1774*/∆*orf19.1117*) strains after 24-h growth in WT *E. faecalis* conditioned media. Data are shown as individual points (n = 6) with lines representing the mean; statistical significance was determined using an unpaired Student’s *t* test (*P* = 0.0018).

Citrate metabolism in *E. faecalis* is known to produce formate, a short-chain fatty acid (SCFA) which is toxic to fungi (34). *C. albicans* encodes three homologs of FDH, an enzyme known to detoxify formate. All three FDH genes (*FDH1*, *orf19.1117*, and *orf19.1774)* were among the top 15 *C. albicans* genes upregulated in the presence of *E. faecalis* in vitro (Fig. 3*A* and Datasets S1 and S3). In fact, *orf19.1117* (544-fold) and *orf19.1774* (272-fold) were the top two *C. albicans* genes upregulated in response to *E. faecalis* in vitro (Fig. 1*B*). In the mouse gut, the *Candida FDH1* gene is also upregulated in the presence of *E. faecalis* (Fig. 1*C*); read counts for the other two FDHs were low and did not pass our filtering thresholds.

FDHs are induced by formate and detoxify formate by oxidizing it to CO_2_ reducing NAD^+^ to NADH. Given the strong upregulation of *C. albicans* FDHs in the presence of *E. faecalis*, we hypothesized that *E. faecalis*-derived formate serves as the inducer for this upregulation. We first tested whether the increase in *E. faecalis* citrate metabolism correlated with an increase in extracellular formate concentrations by measuring formate directly. BHI(-glu) media contains undetectable levels of formate. After 24 h of growth, the supernatant of a WT *E. faecalis* culture in BHI(-glu) contained 0.5 g/L formate (Fig. 3*B*). When BHI(-glu) (which contains low levels of citrate) is supplemented with 0.2% citrate, the formate concentration for WT *E. faecalis* cultures increases to 0.85 g/L (Fig. 3*B*). A ∆*citH/∆citO E. faecalis* strain, which is unable to metabolize citrate, produced significantly lower levels of formate under BHI(-glu,+cit) conditions. *E. faecalis* formate production also requires the pyruvate formate lyase (PFL) enzymes (PflA, PflB); when the genes for these enzymes are knocked out, the ∆*pflA/∆pflB E. faecalis* mutant is unable to produce or secrete formate (Fig. 3*B*).

To test whether the *C. albicans* FDH enzymes are protective against formate, we constructed *C. albicans* mutants deleted for one, two, or all three of the FDH genes. In BHI(-glu) media supplemented with 0.4% formate, the triple mutant (FDH∆3), various double mutants, and the ∆*fdh1* single mutant all showed reduced growth compared to the WT strain (Fig. 3*C*). The deletion of *FDH1* had the most significant impact on growth out of all three FDH homologs.

Given that the FDHs enable *C. albicans* to tolerate formate, we tested whether they provide a growth advantage to *C. albicans* in the presence of *E. faecalis* under conditions where the bacterium is synthesizing formate from citrate. We exposed WT *C. albicans* and the FDH∆3 strain to WT *E. faecalis*-conditioned BHI(-glu,+cit) media for 24 h. The FDH∆3 strain displayed a significant growth defect in the *E. faecalis-*conditioned medium compared to WT *C. albicans* (Fig. 3*D*). In summary, the formate produced by *E. faecalis* citrate metabolism is toxic to *C. albicans,* but its effects are mitigated by the *C. albicans* FDH enzymes, which are highly upregulated in the presence of *E. faecalis*.

## Discussion

Many studies have implicated individual microbial species in various human health outcomes; however, detailed molecular interactions *between* different microbial species within the gut microbiome have not been extensively studied. Evidence to date suggests that the impact of any particular species on host interactions (e.g., drug metabolism, gut epithelial integrity) can be influenced by the interactions with other community members (37, 38). Fully understanding the behavior of a species in the microbiome, therefore, requires unraveling its interactions with other microorganisms of this community. The complexity of the adult microbiome presents significant experimental challenges in studying interactions between different species within this intricate network. In this study, we used the minimally colonized preterm infant gut microbiome as a starting point to study interactions between two common gut colonizers, the yeast *Candida albicans* and the bacterium *Enterococcus faecalis.* We first examined how the presence of each species affects the other’s gene expression profile during growth together in the gut of gnotobiotic mice and in vitro conditions designed to mimic certain features of the gut environment. The results reveal that each species greatly influences the gene expression pattern of the other, offering insights into the large scale of changes induced by coculture. For example, we identified 16 *C. albicans* genes that are highly upregulated in the presence of *E. faecalis*, both in vitro and in the mouse gut, none of which have been studied.

Although the RNA-seq results are valuable in their own right (especially in the comparison between the in vitro condition and the mouse gut), we believe the most significant conclusions of this paper lie in the follow-up experiments where we developed and tested specific hypotheses based on the RNA-seq results. In analyzing the results, genes involved in citrate production and citrate breakdown stood out as being highly induced in *C. albicans* and *E. faecalis,* respectively. On exposure to *E. faecalis*, *C. albicans* upregulated the production of Cit1 (citrate synthase). In those same cocultures, *E. faecalis* greatly increased the expression of genes responsible for citrate breakdown. Competitive growth experiments showed that, in the presence of *C. albicans*, *E. faecalis* strains capable of metabolizing citrate had a growth advantage over otherwise isogenic *E. faecalis* strains deleted for genes needed to metabolize citrate. In other words, *E. faecalis* can utilize citrate produced and secreted by *C. albicans*, effectively exploiting this metabolic interaction to enhance its own fitness. We also showed that glucose depletion is sufficient to upregulate *CIT1* and believe that glucose depletion by *E. faecalis* is the primary signal that triggers the upregulation of *CIT1* under coculture conditions. These findings are highly relevant to a recent study showing that *E. faecalis* strains capable of metabolizing citrate exhibited increased growth in blood and urine, as well as heightened virulence in a *Galleria* infection model (30). Also relevant to our study, past studies have shown that intra- and interspecies cross-feeding of citrate can impact the virulence and fitness of bacteria (39, 40); our work provides an example of cross-kingdom (eukaryote to bacteria) metabolic cross-feeding of citrate.

Our transcriptional profiling also revealed that the *C. albicans* FDH genes (*FDH1*, *orf19.1117*, *orf19.1774*) are highly upregulated in the presence of *E. faecalis*. Formate, a SCFA produced by numerous gut bacteria, is one of the main byproducts of citrate metabolism in *E. faecalis* (41). SCFAs, such as butyrate or acetate, have been widely studied in recent years due to their role in intestinal epithelial integrity and immune modulation (42). While the role of formate in the gut microbiome is less well understood compared to other SCFAs, recent reports indicate that formate concentrations increase in the gut microbiome during dysbiosis in mouse models of colitis and that bacteria encoding FDH enzymes have a fitness advantage in this dysbiotic environment (43).

Based on our RNA-seq data, we developed the following hypothesis regarding formate: In our coculture experiments, *E. faecalis* metabolized citrate (produced by *C. albicans*) to formate, which is subsequently secreted. This secreted formate then induces the expression of three *C. albicans* FDH genes. Formate is known to be toxic to yeast at high concentrations (44, 45), and we proposed that the upregulation of these FDHs is a mechanism by which *C. albicans* detoxifies formate produced by *E. faecalis*. To confirm this hypothesis, we demonstrated that *C. albicans* FDH enzymes confer a growth advantage in the presence of both pure formate and *E. faecalis*-derived formate, as evidenced by the increased fitness of WT *C. albicans* compared to isogenic FDH-deletion strains. Despite its defense mechanisms, it is likely that in the wild, *C. albicans* proliferation is constrained by the formate produced by *E. faecalis*. Thus, in different contexts, the metabolic cycle we describe could be both synergistic and antagonistic.

**Fig. 4.**
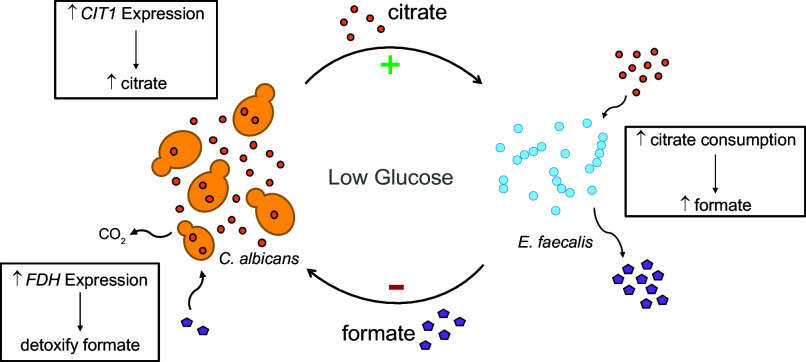
Citrate Cross-feeding Metabolic Cycle between *C. albicans* and *E. faecalis* in coculture. In low-glucose conditions (either in the mouse gut or in vitro after glucose depletion), *C. albicans* upregulates the expression of *CIT1* and increases citrate production and subsequent secretion. In response, *E. faecalis* upregulates the expression of its *cit* operon, which increases citrate consumption. A major byproduct of *E. faecalis* citrate metabolism is formate, which is secreted. The increase in extracellular formate induces the upregulation of *C. albicans* FDH enzymes, which detoxify formate into carbon dioxide.

*C. albicans* and *E. faecalis* are typically low-abundance microbes in the healthy adult gut microbiome, but in periods of dysbiosis, they rise to high concentrations where they have the potential to translocate from the gut to the bloodstream and cause lethal infection (16, 46–48). Collectively, this work demonstrates a circular cross-feeding interaction between this yeast and a bacterium: *C. albicans* upregulates genes to tolerate the toxic byproduct generated by the very metabolic pathway it supports in *E. faecalis* (Fig. 4). Citrate metabolism and formate production are ubiquitous among other resident gut bacteria (49–51); therefore, the metabolic network presented here may serve as a proxy for interactions between *C. albicans* and other bacterial members of the gut microbiome and at other mucosal sites of colonization. Even though *C. albicans* and *E. faecalis* are only distantly related, we show that the two microbes — together — complete a metabolic cycle that can be both mutually beneficial and antagonistic.

## Materials and Methods

### Strains, Media, and Growth Conditions.

*Candida albicans* and *Enterococcus faecalis* strains and media used in this study are detailed in *SI Appendix*, Table S1 and S2. *C. albicans* strain SC5314 was used as the wild-type strain for all experiments and used to derive all mutant strains. *E. faecalis* strain OG1RF was the wild-type and parental strain for all experiments and mutant generation. Strains were maintained as glycerol stocks and stored at −80 C. Unless otherwise stated, in vitro experiments were performed in an Anaerobic Chamber (Coy Lab) set at the following parameters: 0.2% O_2_, 5% CO_2_, ambient air temperature (hereby referred to as “hypoxic chamber”.) All media and consumable reagents (e.g., plates, serological pipettes, pipette tips) were moved into the chamber at least 1 d before the start of experiments to deoxygenate. Strains were struck in ambient air onto BHI agar plates, after which the plates were ported into the chamber and placed in a 37 °C incubator. Experimental liquid cultures were grown in 24 deep-well plates at 37 °C with agitation (800 RPM).

### *C. albicans* Mutant Generation.

Plasmid and primers used in strain construction are detailed in *SI Appendix*, Tables S3 and S4. Gene deletion mutants in *C. albicans* were constructed using a CRISPR-Cas9 method detailed in (52), modified from (53). Instead of 80 bp of homology, only 60 bp of homology directly flanking the start and stop codons were used to construct the repair template.

### *E. faecalis* Mutant Generation.

Primers and plasmids used in strain construction are detailed in *SI Appendix*, Tables S3 and S4. Gene deletion mutants in *E. faecalis* were constructed using the CRISPR-Cas12 method outlined in (54), with some modifications. Briefly, pJC005.em was restriction digested or amplified. Three inserts (pUCsRNAP-gRNA, 650 bp upstream homology to the gene, and 650 bp downstream homology) were amplified via PCR, inserted into the linearized backbone using Gibson Assembly (NEB), and transformed into NEB DHα cells. After confirming via sequencing, the constructed plasmid was transformed into *E. faecalis* OG1RF following the protocol outlined in (55). Todd Hewitt Broth (THB) + 10ug/mL erythromycin agar plates were used for selection posttransformation. All subsequent steps used BHI media and agar plates with added 10ug/mL erythromycin and 250 ng/mL anhydrous tetracycline when needed.

### Cell Growth for In Vitro RNA-Seq.

Single colonies of *C. albicans* and *E. faecalis* (from BHI plates grown for 1 d at 37 °C in hypoxic chamber) were inoculated in 2 ml BHI media (BD Difco) and grown overnight. In the morning, cultures were backdiluted to OD of 0.1 in 6 ml BHI media and grown as above for 4 h. *C. albicans* was backdiluted to an OD600 of 0.2 in BHI media, and *E. faecalis* cells were centrifuged and resuspended in fresh BHI media at an OD600 of 2. Both *C. albicans* and *E. faecalis* in the experimental co- and monocultures were started at an OD of 0.2 in 2 ml BHI media. Experimental cultures were grown at 37 °C for 4 h, after which they were harvested by centrifugation (3,500 RPM for 5 min) and pellets were flash frozen and stored at −80 °C.

### Colonization of Germ-Free Mice Gastrointestinal Tract.

*C. albicans* strains were grown overnight in Yeast Peptone Dextose (YPD) media (2% Bacto peptone, 2% dextrose, 1% yeast extract) at 30 °C and *E. faecalis* strains were grown overnight in BHI media at 37 °C. Cells were washed twice in sterile Phosphate Buffer Saline (PBS) and concentration was determined using a BD Accuri C6 flow cytometer. All animal procedures were approved by the UCSF Institutional Animal Care and Use Committee (protocol #AN201437-00A) and performed in the University of California San Francisco (UCSF) Gnotobiotics Core Facility. Mice were kept in cages contained within a sterile isolator for the duration of the experiment. A minimum of 8 germ-free C57BL/6 mice of mixed sex were colonized via gavage with i) 3 × 10^6^ *C. albicans* cells, ii) 3 × 10^6^ *C. albicans* cells and 5 × 10^8^ *E. faecalis* cells, or iii) 5 × 10 ^8^ *E. faecalis* cells in 0.1 mL PBS. At the end of 10 d, mice were euthanized, and the cecum contents were added to 5 mL RNAlater stabilization solution (Invitrogen AM7020), and placed at 4 °C. After 1 d at 4 °C, cecum contents-RNAlater slurries were aliquoted, centrifuged, and the RNAlater supernatant was removed. Cecum content pellets were stored in −80 °C until used for downstream processing.

### Plating of Fecal Pellets to Determine Microbial Burden.

Colonization of gnotobiotic mice was monitored by the collection and plating of fecal pellets at three and 10 d postgavage. The wet weight of a fecal pellet was recorded. Pellet was then homogenized in 500µL sterile PBS via vortexing. PBS-fecal slurry was serially diluted and plated on YPD + 250 µg/mL erythromycin (yeast-selection) or BHI + 50 ug/mL nystatin (bacteria-selection). Yeast plates were grown at 30 °C and bacteria plates were grown at 37 °C overnight. Colony-forming units (CFUs) were enumerated and calculated relative to the starting weight of the pellet to determine CFU/g fecal pellet.

### RNA Extraction from In Vitro Samples and Cecum Contents.

RNA was extracted from cecal contents using a protocol modified from (56) that was optimized to yield high purity and quantity RNA from gnotobiotic mice. Full protocol can be found in (57)

For in vitro samples, RNA was extracted using the same protocol with few modifications: Only 1 additional phenol-chloroform extraction was performed prior to isopropanol precipitation. For all RNA samples, concentration of total RNA was determined using a Nanodrop and RNA integrity was analyzed using the Tapestation High-Sensitivity RNA kit (Agilent 5067-5579).

### rRNA Depletion and Library Preparation.

rRNA was depleted from total RNA using filamentous-fungi riboPOOL, Pan-Bacteria riboPOOL, and Mus musculus riboPOOL (siTOOLs) using the manufacturer’s protocol with some modifications. For dual-species in vitro samples, bacteria and fungi riboPOOL probes were mixed at a 3:1 ratio. For dual-species cecum content samples, probes were mixed at an 8:1:1 bacteria:fungi:mouse ratio. For all single species samples, the probes for the specific species were used alone. 1.5 to 2 µg total RNA was used as input. The remaining RNA was concentrated using Zymo clean & concentrator-5. RNA concentration was determined using the Qubit High-Sensitivity RNA kit and success of depletion was determined using the Tapestation High-Sensitivity RNA kit (Agilent 5067-5579).

Total RNA-seq libraries were created using the CORALL Total RNA-seq V1 kit (Lexogen 095) according to manufacturer protocol. 5 ng of rRNA-depleted RNA was used as input into library generation. Concentration and average size of resulting DNA library determined using the Tapestation High-Sensitivity DNA kit (Agilent 5067-5584). Libraries were pooled in equimolar amounts and sequenced at the UCSF CAT on the Illumina NextSeq Platform with the following parameters: 100 bp single-end reads with an average of 45 million reads per sample.

RNA-seq reads were filtered for quality and trimmed of Illumina sequencing adapters and polyA tails using Fastp (0.20.1) (58). FastQC (0.11.9) was used to perform quality checks of data (59). Reads were mapped using STAR (2.7.9a) (60). *C. albicans* monocultures were mapped to the *C. albicans* SC5314 Assembly 22 (version A22-s07-m01-r105) chromosomes file from Candida Genome Database (CGD) (61), modified to contain only A allele chromosomes and novel transcripts found in (62). *E. faecalis* monocultures were mapped to the *E. faecalis* OG1RF genome (ASM17257v2, assembly 46) from EnsembleBacteria (63). Reads from coculture samples were mapped simultaneously onto a concatenated *C. albicans*-*E. faecalis* genome. After alignment, resulting BAM files were filtered using Samtools (1.14) to discard any reads less than 30 bp long (64). These reads, despite being a small proportion of the total mapped reads (<3%) were found to erroneously map and create false positives in downstream results, especially in otherwise lowly expressed genes. Read counts for each species were then extracted using Subread FeatureCounts (2.0.1) using the appropriate genome version specific GFF file (65). Count normalization and differential expression analysis was performed using DESeq2 (1.40.2) (66). Mouse and in vitro samples were loaded and processed as separate DESeq objects. Counts tables were filtered after generating DESeq objects to only contain genes with more than 10 raw counts in at least 3 samples (in vitro) or 8 mice. Log_2_ Fold Change values were calculated using DESeq2 *lfcShrink* function, using “ashr” for LFC shrinkage (67).

### Gene Expression Measurements via RT-qPCR.

To measure the gene expression of various genes in either *C. albicans* or *E. faecalis*, mono- or cocultures were grown as outlined in the in vitro RNA-seq section of the methods above. Overnight and outgrowth cultures were grown in BHI media (Alpha Biosciences) with added 0.2% sodium citrate. Media used in experimental cultures is detailed in *SI Appendix*, Table S1 with or without added 0.2% sodium citrate. Total RNA was extracted from frozen cell pellets following the RNA-extraction protocol detailed above with modifications: 1) only 1 phenol-chloroform extraction was performed prior to isopropanol precipitation, 2) no column clean-up was used (postprecipitation RNA was used directly in DNase treatment) and 3) no lithium chloride precipitation was used. RNA concentration was measured using a nanodrop and RNA integrity was measured using Tapestation High Sensitivity RNA kit. cDNA was generated from 450 ng of total RNA using LunaScript^®^ RT SuperMix Kit (New England Biolabs E3010). RT-qPCR was performed with iTaq Universal SYBR Green Supermix (Biorad 1725124) in 5 µL reaction volumes with 1µL of cDNA (diluted 1:4 before use). Primers listed in *SI Appendix*, Table S5 were used at a final concentration of 0.3 µM. A minimum of 3 technical replicates was measured per sample–primer pair.

The in vitro RNA-seq expression data generated in this study was used for *C. albicans* housekeeping gene selection. Two housekeeping genes were selected per species based on the following criteria: 1) Log_2_ Fold Change in expression between coculture and monoculture between −0.25 and 0.25, 2) annotated role in core, essential processes (e.g. DNA replication, cell cycle machinery, RNA polymerase subunits), 3) one lowly expressed and one highly expressed gene. Based on these criteria, *C. albicans* housekeeping genes used in this study were *ORF19.6843* and *IRR1*. Results were analyzed using the ∆∆Ct method, with Ct values for each sample normalized to the average Ct value of the two housekeeping genes.

### Citrate Measurements.

Supernatants of samples grown for qPCR assays were filter-sterilized with a 0.2 µM polyethersulfone (PES) filters and flash-frozen using liquid nitrogen until use in downstream assays. Citrate concentrations were measured using Citric Acid Kit (Megazyme K-CITR) following the manufacturer’s microplate assay procedure using ½ volume reactions. Absorbance (340 nM) was measured using a Tecan Spark 10 M.

### *E. faecalis* Growth Curves with or without Citrate in Hypoxic Conditions.

Single colonies of *E. faecalis* strains (from BHI plates grown at 37 °C for 1 d in a hypoxic chamber) were used to inoculate 2 ml of BHI media (Alpha Biosciences) with added 0.2% sodium citrate. Cultures were washed twice and resuspended in PBS, and backdiluted into 100 μL of various BHI media (detailed in *SI Appendix*, Table S1) with or without added 0.2% sodium citrate at a final OD600 of 0.01 in a flat-bottom, clear 96-well plate. 100µL of each medium was included on the plate as a blank control. OD600 of each well was measured every 15 min with shaking between each time point using a Tecan Infinite Nano M Plus in the hypoxic chamber. At each time point, the absorbance value of each well was corrected by subtracting the absorbance value of the corresponding blank media well.

### *E. faecalis* Growth Assays in the Presence of *C. albicans* in Hypoxic Conditions.

Single colonies of *E. faecalis* or *C. albicans* strains (from BHI agar plates grown at 37 °C for 1 d in hypoxic chamber) were used to inoculate 2 mL of BHI media (Alpha Biosciences) with added 0.2% sodium citrate and grown overnight. Cultures were washed twice and resuspended in PBS. Experimental cultures were inoculated into 2 mL various BHI media (detailed in Table 2.2) with or without added 0.2% sodium citrate at a starting OD600 of 0.1 for *C. albicans* and 0.01 for *E. faecalis*. Cultures were grown at 37 °C with agitation for 24 h. Aliquots from 24-h cultures were 10-fold serially diluted and 100 μL of the 10^6^ dilution was plated on BHI + 50 μg/mL nystatin plates to select for *E. faecalis*. Colonies were enumerated and used to calculate CFU/mL of cultures.

### Formate Measurements.

Supernatants of cultures were filter-sterilized with 0.2 µM Polyethersulfone (PES) filters and flash-frozen using liquid nitrogen until used in downstream assays. Formate concentrations were measured using Formic Acid Assay Kit (Megazyme K-FORM) following the manufacturer’s microplate assay procedure using ½ volume reactions. Absorbance (340 nm) was measured using a Tecan Spark 10 M.

### *C. albicans* Growth Curve in Formate.

Single colonies of *C. albicans* WT and single, double, and triple deletions of FDH genes (see *SI Appendix*, Table S2 for details) were inoculated into 3 mL YPD overnight at 30 °C. In the morning, 1 mL cells were centrifuged at 5,000 RPM for 1 min and washed twice in 1 mL sterile water. Cells were diluted to a final OD600 of 0.005 in 100 µL of various media (see *SI Appendix*, Table S1 for details) with or without the addition of 4 or 0.8% formate in a flat-bottomed 96-well plate. 100µL of each medium was included on the plate as a blank control. OD600 of each well was measured every 15 min with shaking between each time point using a Tecan Spark 10 M in aerobic conditions. At each time point, the absorbance value of each well was corrected by subtracting the absorbance value of the corresponding blank media well.

### *C. albicans* Growth in WT *E. faecalis* Conditioned Media.

*E. faecalis* conditioned medium was prepared as follows: *E. faecalis* was inoculated into BHI(-glu,+cit) media and grown overnight at 37 °C, 800RPM in hypoxic conditions. In the morning, the culture was centrifuged to pellet the cells and the supernatant was filter-sterilized using a PES 0.22uM filter. Supernatant was then lyophilized overnight. After lyophilization, the lyophilized medium was reconstituted to 1X concentration with fresh, deoxygenated BHI(-glu) in the hypoxic chamber.

Single colonies of *C. albicans* WT and FDH∆3 (triple FDH deletion mutant) (from BHI agar plates grown at 37 °C for 1 d in hypoxic chamber) were used to inoculate 2 mL of BHI media (Alpha Biosciences) with added 0.2% sodium citrate and grown overnight. In the morning, cells were backdiluted to an OD of 0.1 in fresh BHI(+glu,+cit) and allowed to grow for 3.5 h in 37 °C, hypoxic conditions. Cells were spun down and resuspended in BHI(-glu) media. *C. albicans* cells were diluted into the *E. faecalis* conditioned medium at a final OD of 0.005 in a deep, 24-well plate and grown at 37 °C, hypoxic conditions for 24 h. After 24 h, serial dilutions were made in sterile PBS and 100 μL of multiple dilutions were plated for single colonies on YPD agar plates.

## Supplementary Material

Appendix 01 (PDF)

Dataset S01 (XLSX)

Dataset S02 (XLSX)

Dataset S03 (XLSX)

## Data Availability

Raw RNA-seq reads (fastq), Aligned BAM files, and gene count tables have been deposited into Gene Expression Omnibus (GSE288745) (68).
