# Supplementary material for: Shared metabolism between a bacterial and fungal species that reside in the human gut
Source: Proc Natl Acad Sci U S A. 2025 Aug 25;122(35):e2504785122. doi: 10.1073/pnas.2504785122 (PMC12415286; doi:10.1073/pnas.2504785122)
Supplement: Supplementary file 1 — Appendix 01 (PDF) [file pnas.2504785122.sapp.pdf]

Supporting Information for

Shared Metabolism Between a Bacterial and Fungal Species that Reside in the Human Gut

Haley Gause[a], Alexander D. Johnson[a,b]

[a]TETRAD Graduate Program, Department of Biochemistry and Biophysics, University of California, San Francisco, CA 94158

[b]Department of Microbiology and Immunology, University of California, San Francisco, CA 94158

Corresponding Author: Alexander D. Johnson

Email: ajohnson@cgl.ucsf.edu

This PDF file includes:

Tables S1 to S5
Figures S1 to S5
SI References

Other supporting materials for this manuscript include the following:

Datasets S1 to S3

Table S1: ***C. albicans*** **and** ***E. faecalis*** **strains used in this study**

C. albicans Strains				
Strain Name	Description/Informal Name	Genotype	Parent	Source
SC5314	WT	WT	—	
yHG140	Δ*cit1*	*cit1Δ/cit1Δ*	SC5314	This study
yHG146	Δ*fdh1*	*fdh1Δ/fdh1Δ*	SC5314	This study
yHG169	Δ*orf19.1117*	*orf19.1117Δ/orf19.1117Δ*	SC5314	This study
yHG135	Δ*orf19.1774*	*orf19.1774Δ/orf19.1774Δ*	SC5314	This study
yHG153	Δ*fdh1*/Δ*orf19.1774*	*fdh1Δ/fdh1Δ; orf19.1774Δ/orf19.1774Δ*	yHG146	This study
yHG154	Δ*orf19.1774*/Δ*fdh1*	*orf19.1774Δ/orf19.1774Δ; fdh1Δ/fdh1Δ*	yHG135	This study
yHG181	Δ*orf19.1117*/Δ*orf19.1774*	*orf19.1117Δ/orf19.1117Δ; orf19.1774Δ/orf19.1774Δ*	yHG169	This study
yHG183	Δ*fdh1*/Δ*orf19.1117*	*fdh1Δ/fdh1Δ; orf19.1117Δ/orf19.1117Δ*	yHG146	This study
yHG171	*FDHΔ3 isolate 1 (Δfdh1/Δorf19.1774/ Δorf19.1117)*	*fdh1Δ/fdh1Δ; orf19.1774Δ/orf19.1774Δ; orf19.1117Δ/ orf19.1117Δ*	yHG153	This study
yHG173	*FDHΔ3 isolate 2 (Δorf19.1774/Δfdh1 Δorf19.1117)*	*orf19.1774Δ/orf19.1774Δ; fdh1Δ/fdh1Δ; orf19.1117Δ/ orf19.1117Δ*	yHG154	This study
E. faecalis Strains				
Strain Name	Description/Informal Name	Genotype	Parent	Source
OG1RF	WT	WT	–	(1)
bHG35	Δ*citHO*	Δ*citH*;Δ*citO*	OG1RF	This study
bHG39	ΔpflAB	Δ*pflA*;Δ*pflB*	OG1RF	This study

Table S2: Media Used in this Study
*Lack of citrate is not denoted by manufacturer, but was empirically determined using Citrate Acid Kit (see methods)

Media Name	Source	Nutrient Description (from manufacturer)	Assay Used In
BHI Difco	BD Difco™ 237500	0.2% dextrose	RNA-seq
BHI	Alpha Biosciences (B02-113-500)	0.2% dextrose, no citrate*	Growth assays, qPCR, citrate and formate measurements
BHI-glucose (BHI(-glu))	Alpha Biosciences (B02-114-500)	No added dextrose, no citrate*	Growth assays, qPCR, citrate and formate measurements

Table S3: Plasmids

Name	Description	Used in	Source
pADH34	SAT1-Knock Out dDNA Template	*C. albicans* mutant strain construction	(2)
pADH143	Knock Out gRNA Template	*C. albicans* mutant strain construction	(3)
pp2280	P_{ENO1}-Cas9-T_{CYC1} construct	*C. albicans* mutant strain construction	(4)
pJC005.em	(Empty Backbone) CRISPR-Cas12a genome editing of E. faecium	*E. faecalis* mutant strain plasmid construction	Gifted from James Collins (5) (Addgene # 182738)
pUCsRNAP	Swapping in small RNA promoter, 23-bp spacer sequence, & 19-bp repeats into pJC005 plasmids	*E. faecalis* mutant strain plasmid construction	Gifted from James Collins (5) (Addgene # 182746)
pHG3	pJC005.em backbone; insert: gRNA(citH)-citH upstream homology-citO downstream homology	*E. faecalis* mutant strain construction	This study
pHG7	pJC005.em backbone; insert: gRNA(pflB)-pflB upstream homology-pflA downstream homology	*E. faecalis* mutant strain construction	This study

Table S4: Primers Used in Strain Construction
Bolded bases in primer sequences denote the gRNA used in CRISPR strain construction

Primer Name	Primer Sequence	Used for
oHG85_PSNR52_F	catctaatcaactcccagat	*C. albicans* Mutant Generation – PSNR fragment 1
oHG86_PSNR52_R	caaattaaaaatagtttacgcaa	*C. albicans* Mutant Generation – PSNR fragment 1
oHG212_CIT1_gRNA_F	cttgcgtaaactatttttaatttg**aaacaattcaaaaaagaaca**gttttagagctagaaatagca	*C. albicans* Δ*cit1* Generation – PSNR fragment 2
oHG88_gRNA-scaffoldTerminator_R	taaaaaaactcgagaaaaaaagcac	*C. albicans* Δ*cit1* Generation – PSNR fragment 2
oHG210_CIT1_downhomology_SAT1_R	atcattgaataataaataataataaataaaatctagtcaaacaggatacccgtcaaatcacgctctagaactagtggatc	*C. albicans* Δ*cit1* Generation – homology flanked repair template
oHG211_CIT1_uphomology_SAT1_F	tcttcttttccttttcccatcttttaagtttcttttagaatatagtatatttatcaaacactagtgaattcgcgctcgag	*C. albicans* Δ*cit1* Generation – homology flanked repair template
oHG208_CIT1_orfcheck_R	ctgtaaatcttagcagcaatgg	*C. albicans* Δ*cit1* Generation – ORF check
oHG209_CIT1_orfcheck_F	gggaaggttctgttttggac	*C. albicans* Δ*cit1* Generation – ORF check
oHG211_CIT1_uphomology_SAT1_F	tcttcttttccttttcccatcttttaagtttcttttagaatatagtatatttatcaaacactagtgaattcgcgctcgag	*C. albicans* Δ*cit1* Generation – flank check
oHG226_CIT1_downflankcheck_R	agagcaccaccaatagtttc	*C. albicans* Δ*cit1* Generation – flank check
oHG185_RNAP_F	gaattcgtcataatctttaatttgaaaagatttaaggc	*E. faecalis* Mutant Generation – RNA promoter
oHG186_RNAP-F_citHgRNA1	acaagagtagaaatt**tcattggatcgcctttagcaaag**atctacaagagtagaaattatggtggaatg	*E. faecalis* Mutant Generation – RNA promoter-gRNA(*citH*)
oHG187_citH_upstream_F	gagcatatggatatgaatttctactcttgtagattctggaaactctcctgctg	*E. faecalis* Mutant Generation – citH upstream homology
oHG188_citH_upstream_R	gtttttcttcctttctttttagaactttttg	*E. faecalis* Mutant Generation – citH upstream homology
oHG207_citO_downstream_F	aaaaagaaaggaagaaaaacgcaaaaaagacctgcagtcg	*E. faecalis* Mutant Generation – citO downstream homology
oHG192_citO_downstreamflank_R	gcatgtctgcaggcctcgaggatggttactacttagtccaaatg	*E. faecalis* Mutant Generation – citO downstream homology
oHG219_citHflanklong_F	gcaacttcttttgcttctgg	*E. faecalis* Mutant Generation – citHO KO confirmation
oHG192_citO_downstreamflank_R	gcatgtctgcaggcctcgaggatggttactacttagtccaaatg	*E. faecalis* Mutant Generation – citHO KO confirmation
oHG310_BstXIRD_RNAP_F	aataaaaagatgccagtgtggtgctggaattcgtcataatctttaatttg	*E. faecalis* Mutant Generation – RNA promoter
oHG311_pflB_gRNA_R	aatt**gcttgttctccatttgtcgcctt**atctacaagagtagaaattatggtggaatgataagggtt	*E. faecalis* Mutant Generation – RNA promoter-gRNA(*pflB*)
oHG312_pflB_uphomology_F	caaatggagaacaagcaatttctactcttgtagatcaccgattgtcatcgttacc	*E. faecalis* Mutant Generation – pflB upstream homology
oHG195_pflB_upstreamflank_R	gaagtgtttgcctccttagtt	*E. faecalis* Mutant Generation – pflB upstream homology
oHG313_pflA_downflank_F	aactaaggaggcaaacacttccaaacgaaaaaatagaaaagaagac	*E. faecalis* Mutant Generation – pflA downstream homology
oHG314_pflA_downhomology_BstXIRD_R	cgatgacgaattccagcacagtcttcttgtgtgcaagttggtg	*E. faecalis* Mutant Generation – pflA downstream homology

Primer Name	Primer Sequence	Used for
oHG315_pflB_flankcheck_F	caagtaatggaagtccacgagatg	*E. faecalis* Mutant Generation – pflAB KO confirmation
oHG316_pflA_flankcheck_R	gcatattgtgtttcttcacttggtg	*E. faecalis* Mutant Generation – pflAB KO confirmation
oHG300_orf19.1117_uphomologySAT1_fwd	gatatctctatttgattgataaaaataacaacttcaacaactactacaacaacaacttaacaacacaaaactagtgaattcgcgctcgag	*C. albicans* Δ*orf19.1117* Generation – homology flanked repair template
oHG301_orf19.1117_downhomologySAT1_rev	gatatttatttaaaaatcaacactataaataaataaaaaacactaatcacataaagatcattgaggttgccgctctagaactagtggatc	*C. albicans* Δ*orf19.1117* Generation – homology flanked repair template
oHG329_orf19.1117_gRNA_primer	cttgcgtaaactatttttaatttg**ggtcaacagtccattctaag**gttttagagctagaaatagca	*C. albicans* Δ*orf19.1117* Generation – PSNR fragment 2
oHG303_orf19.1774_uphomologySAT1_fwd	attgatataacttcattgaattaacaaaccaacaactataataacaactttgacaaacaaacaagcaaaactagtgaattcgcgctcgag	*C. albicans* Δ*orf19.1774* Generation – homology flanked repair template
oHG304_orf19.1774_downhomologySAT1_rev	aatagataaataatactatgaatgaagtgtaaaactagccacctttgaggtatttttggttgttgttagccgctctagaactagtggatc	*C. albicans* Δ*orf19.1774* Generation – homology flanked repair template
oHG305_orf19.1774_gRNA_primer	cttgcgtaaactatttttaatttg**agaatccagattattaggta**gttttagagctagaaatagca	*C. albicans* Δ*orf19.1774* Generation – PSNR fragment 2
oHG306_FDH1_uphomologySAT1_fwd	atattaattgattgacaactacaactttatatcaaaccaactaaatcaaaaagcttaacgaaaaacaaaactagtgaattcgcgctcgag	*C. albicans* Δ*FDH1* Generation – homology flanked repair template
oHG307_FDH1_downhomologySAT1_rev	accgtgagatacataacgtttttagctaaaaaacaaagaaacataatcaaacaagccagctaggattaccgctctagaactagtggatc	*C. albicans* Δ*FDH1* Generation – homology flanked repair template
oHG308_FDH1_gRNA_primer	cttgcgtaaactatttttaatttg**attggttactactactgata**gttttagagctagaaatagca	*C. albicans* Δ*FDH1* Generation – PSNR fragment 2

Table S5: Primers Used in RT-qPCR Assays

Primer Name	Primer Sequence	RT-qPCR Target
oHG238_cit1_qpcr_F	CTGACGCCCAAACTAAGGCT	*C. albicans CIT1*
oHG239_cit1_qpcr_R	TTGGGTGCAAGTGAGATGGA	*C. albicans CIT1*
oHG76_IRR1_qpcr_F	TCTTGCCTGCTTTGACATGC	*C. albicans IRR1*
oHG77_IRR1_qpcr_R	GCGCTGTTCGTCCTTTTCAA	*C. albicans IRR1*
oHG72_orf19.6843_qpcr_F	ACGGAGATGGGGAGGAAGAA	*C. albicans ORF19.6843*
oHG73_orf19.6843_qpcr_R	TCCCAAGCAACCTCAAAATCAC	*C. albicans ORF19.6843*

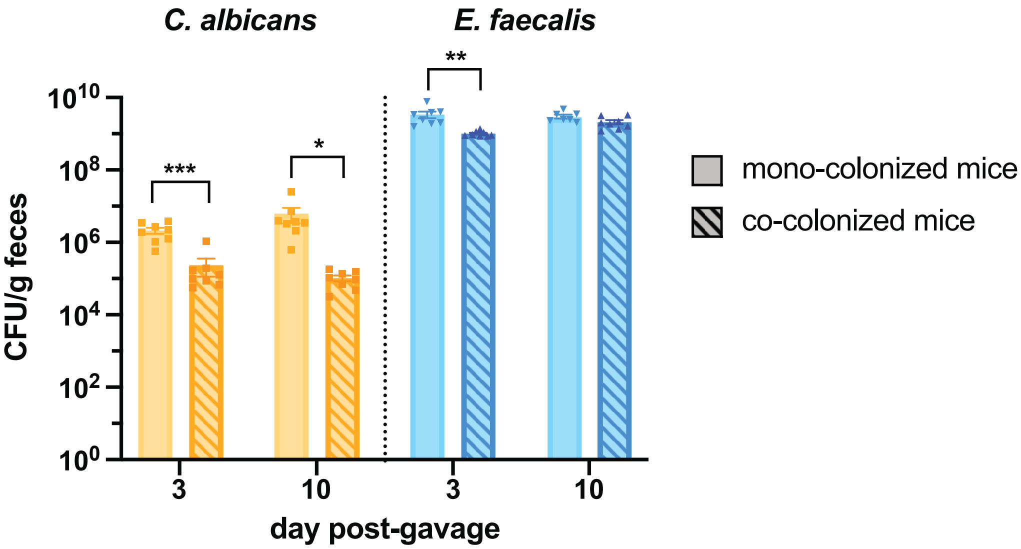

Fig. S1. Microbial burden of gnotobiotic mice mono-colonized or co-colonized with *C. albicans* and *E. faecalis*. Concentration (CFU/g feces) of *C. albicans* and *E. faecalis* in fecal pellets of mice collected on day 3 or day 10 post-gavage. Data are shown as individual points representing a single mouse (n=8), with bars representing the mean and error bars representing ± SEM; statistical significance was determined using an unpaired Student's t-test for each species within each time point (*: $p < 0.05$, **: $p < 0.01$, ***: $p < 0.001$).

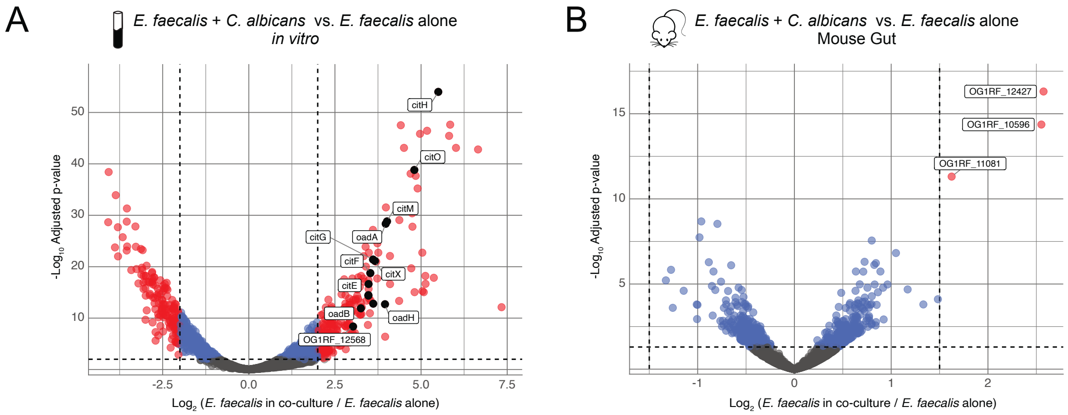

Fig. S2. Transcriptional response of *E. faecalis* to *C. albicans* in co-culture compared to mono-culture. Volcano plots showing *E. faecalis* gene expression changes when co-cultured with *C. albicans* compared to mono-culture *in vitro* (A) or in the mouse gut (B). Each point represents a single *E. faecalis* gene. Induced genes are on the right, and repressed genes are on the left. Genes are color-coded as follows: red: genes with > 4 (*in vitro*) or 3 (mouse) fold-change in expression (adjusted p-value < 0.05); blue: adjusted p-value <0.05, but < 4 (*in vitro*) or 3 (mouse) fold-change in expression; grey: non-significant changes. A) black, labeled dots represent the genes of the *cit* operon responsible for citrate metabolism. B) genes passing fold-change thresholds (> 3-fold change) are labeled.

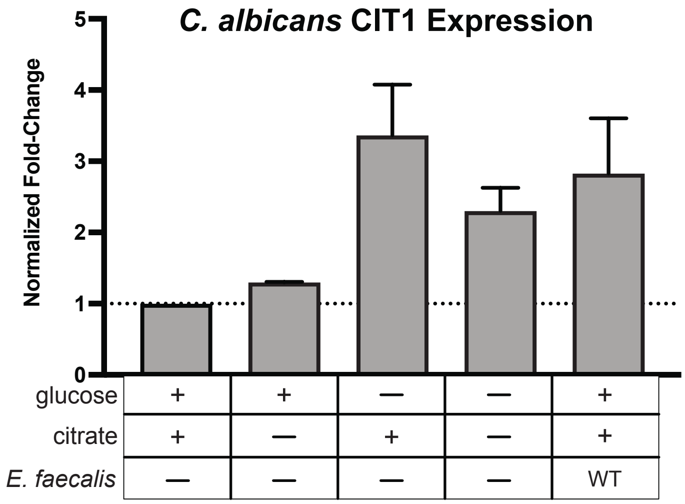

Fig. S3. *C. albicans* upregulates *CIT1* Expression in response to low glucose. RT-qPCR results for *C. albicans CIT1* expression in BHI media with different glucose and citrate availabilities, and in the presence of *E. faecalis*, as indicated by the table below the graph. Data shown as the relative fold-change in expression compared to BHI(+glu,+cit) condition. Bars represent mean + SEM (n=3).

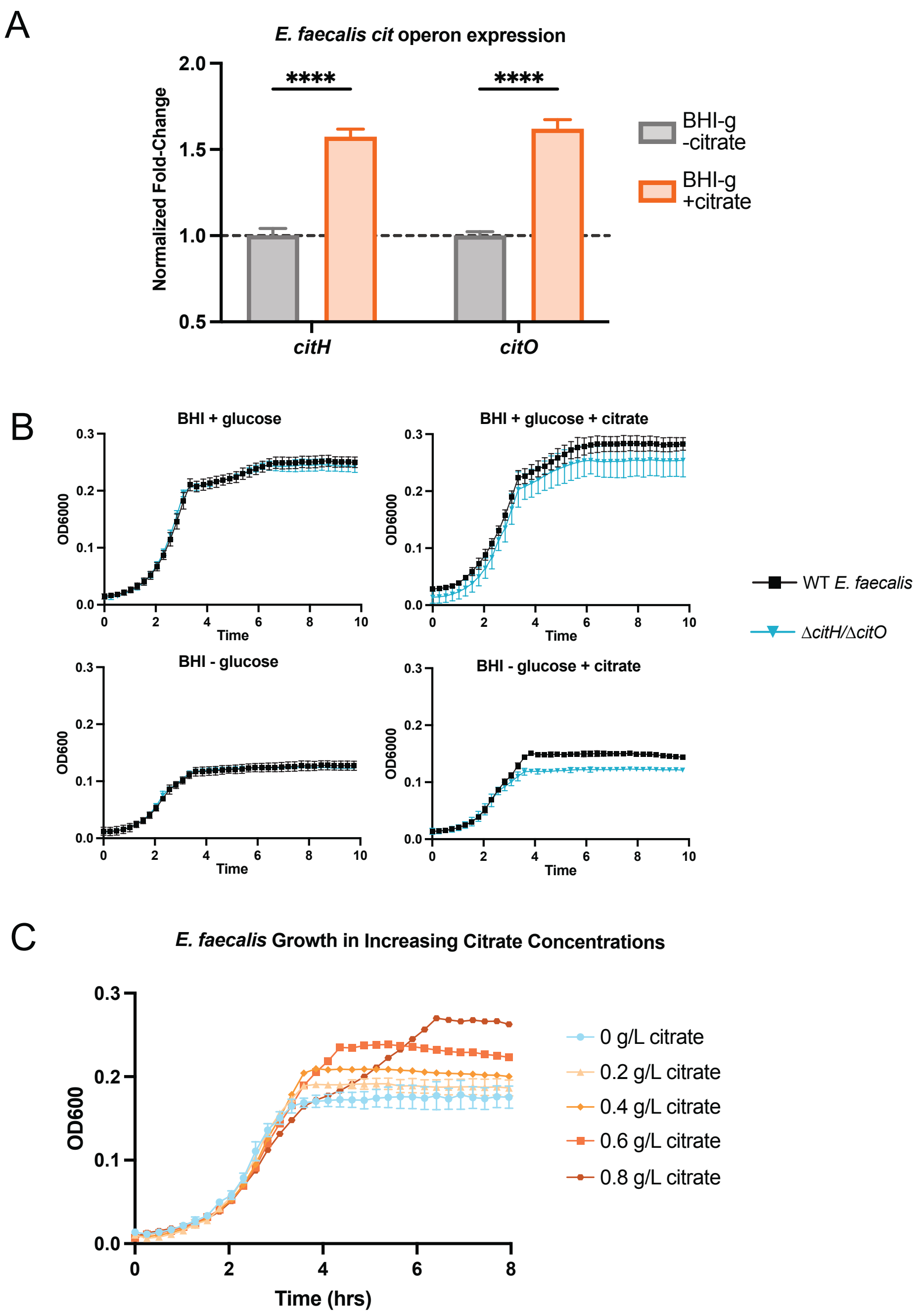

Fig. S4. Citrate metabolism enhances *E. faecalis* growth. A) RT-qPCR data showing change in expression of *citH* and *citO* in *E. faecalis* when grown in citrate-replete media (BHI(-glu,+cit)) relative to citrate deplete media (BHI(-glu)). Bars represent mean ± SEM (n=3). B) Growth curves of WT or Δ*citH/*Δ*citO E. faecalis* when grown in BHI media, 1) with or without 0.2% glucose, and 2) with or without 0.2% citrate. Growth curves measured on Tecan via absorbance (OD600) in 0.2% oxygen, 5% CO_2, 37°C. Each point represents the mean ± SEM (n=3). C) Growth of WT *E. faecalis* scales with concentration of citrate present in media. WT *E. faecalis* was grown in BHI(-glu) with citrate added at increasing concentrations (0 – 0.8 g/L). Growth curves measured on Tecan via absorbance (OD600) in 0.2% oxygen, 5% CO_2, 37°C. Each point represents the mean ± SEM (n=3).

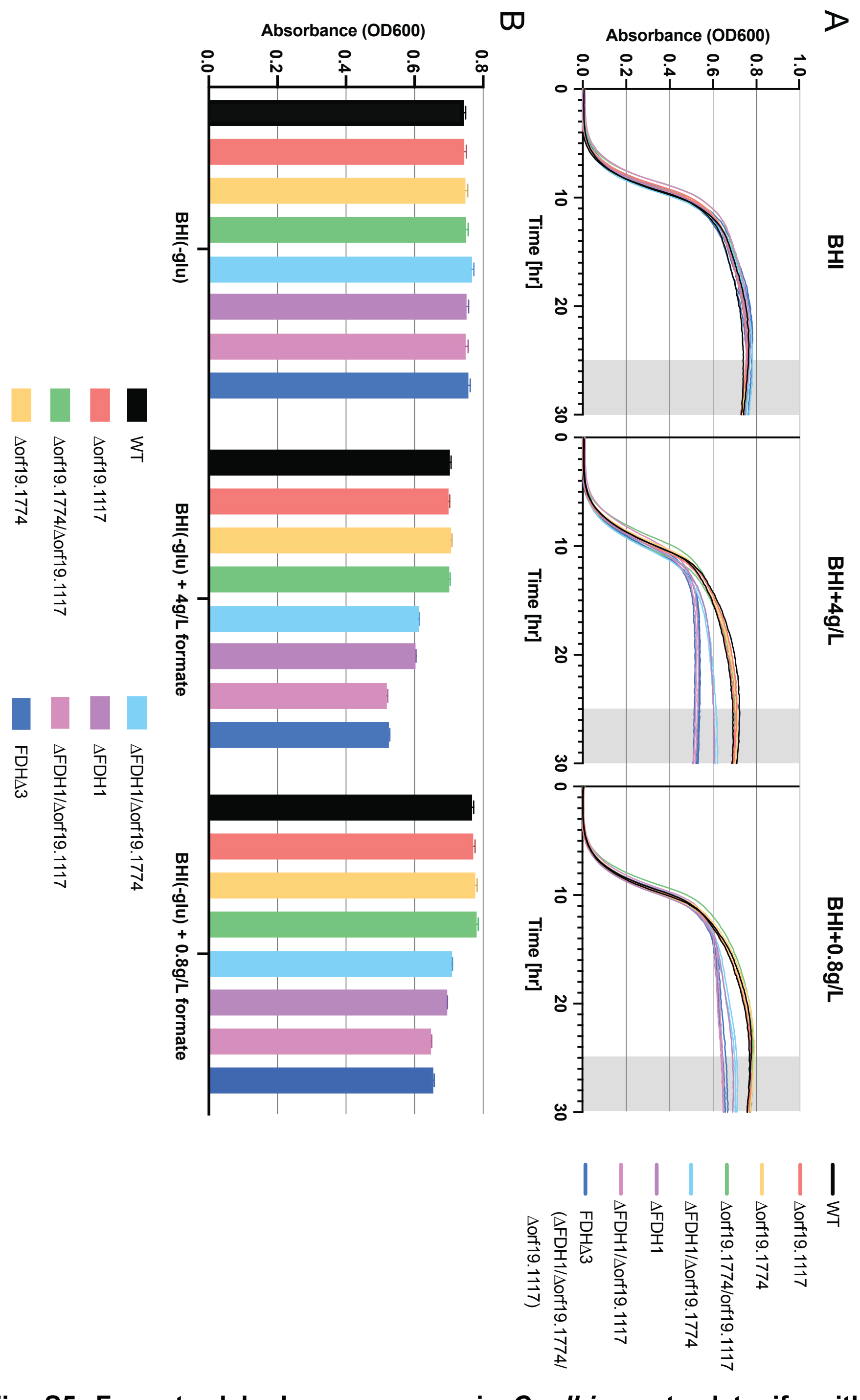

Fig. S5. Formate dehydrogenases equip *C. albicans* to detoxify with *E. faecalis*-derived formate. Growth of *C. albicans* WT and FDH single, double and triple deletion strains grown in BHI(-glu) (left), BHI(-glu) + 4 g/L formate (middle), and BHI(-glu) + 0.8 g/L formate (right). A) Growth curves measured on Tecan via absorbance (OD600) in aerobic conditions, 30 °C. Strains colored according to the legend. Data points within shadowed box (25-30 hours) were averaged to determine OD600 of stationary culture in panel B. B) Absorbance of *C. albicans* cultures at stationary phase, as determined by the average of the OD600 between 25 and 30 hours of growth. Bars are colored according to the legend. Bars represent the mean + SEM across two or four (FDHΔ3) biological replicates.

SI References:

1. B. E. Murray, *et al.*, Generation of restriction map of Enterococcus faecalis OG1 and investigation of growth requirements and regions encoding biosynthetic function. *J. Bacteriol.* **175**, 5216–5223 (1993).
2. A. D. Hernday, S. M. Noble, Q. M. Mitrovich, A. D. Johnson, Chapter 31 Genetics and Molecular Biology in Candida albicans. *Methods Enzym.* **470**, 737–758 (2010).
3. N. Nguyen, M. M. F. Quail, A. D. Hernday, An Efficient, Rapid, and Recyclable System for CRISPR-Mediated Genome Editing in Candida albicans. *mSphere* **2**, 10.1128/mspheredirect.00149-17 (2017).
4. C. Lee, A. Johnson, CRISPR/Cas9 genome editing in Candida albicans. *Protocols.io* (2024).
5. M. J. Chua, J. Collins, Rapid, Efficient, and Cost-Effective Gene Editing of Enterococcus faecium with CRISPR-Cas12a. *Microbiol. Spectr.* **10**, e02427-21 (2022).
